# Basal Cell Carcinoma in Type 2 Segmental Darier's Disease

**DOI:** 10.1155/2012/839561

**Published:** 2011-07-31

**Authors:** Lynne Robertson, Maxwell B. Sauder

**Affiliations:** ^1^Division of Dermatology, Department of Medicine, University of Calgary, Calgary, AB, Canada; ^2^Division of Dermatology, Department of Medicine, University of Ottawa, Ottawa, ON, Canada

## Abstract

*Background*. Darier's disease (DD), also known as Keratosis Follicularis or Darier-White disease, is a rare disorder of keratinization. DD can present as a generalized autosomal dominant condition as well as a localized or segmental postzygotic condition (Vázquez et al., 2002). Clinical features of DD include greasy, warty papules and plaques on seborrheic areas, dystrophic nails, palmo-plantar pits, and papules on the dorsum of the hands and feet. *Objective*. We report a case of basal cell carcinoma developing in a patient with type 2 segmental DD. *Conclusion*. According to the current literature, Type 2 segmental disease is a rare presentation of Darier's disease with only 8 previous cases reported to date. In addition, nonmelanoma skin cancer (NMSC) arising from DD is rarely reported; however, there may be an association between DD and risk of carcinogenesis.

## 1. Introduction

Darier's disease (DD), also known as Keratosis Follicularis or Darier-White disease, is a rare disorder of keratinization. The disease is caused by a loss-of-function mutation in the ATP2A2 gene on chromosome 12q23-24 that encodes the sarco/endoplasmic reticulum calcium ATPase (SERCA2). This loss of function leads to a disruption of Ca^2+^ homeostasis within the keratinocytes,specifically depletion of Ca^2+^ stores in endoplasmic reticulum. Ultimately, the mutation leads to impaired cell-to-cell adhesion with the common histological findings of suprabasal acantholysis and dyskeratosis of cells in the epidermis [1]. DD can present as a generalized autosomal dominant condition as well as a localized or segmental postzygotic condition [2]. Clinical features of DD include greasy, warty papules and plaques on seborrheic areas, dystrophic nails, palmo-plantar pits, and papules on the dorsum of the hands and feet.

## 2. Observation

A 34-year-old woman with type 2 skin presented to clinic for evaluation of a lesion in the left popliteal fossa that had been present for approximately 1.5 years and frequently bled. 

Past medical history was significant for DD which was diagnosed at 12 years of age. This was initially localized to the left side of the body but over the previous ten years had gradually become bilateral. She managed her disease with only emollients and sun protection but as a teenager had tried topical steroids, topical retinoids, and a short course of accutane. She was otherwise well and not on any medication. She denied a past history of excess sun exposure or blistering burns. Neither of her two children or parents were affected by DD. 

**Figure 1:**
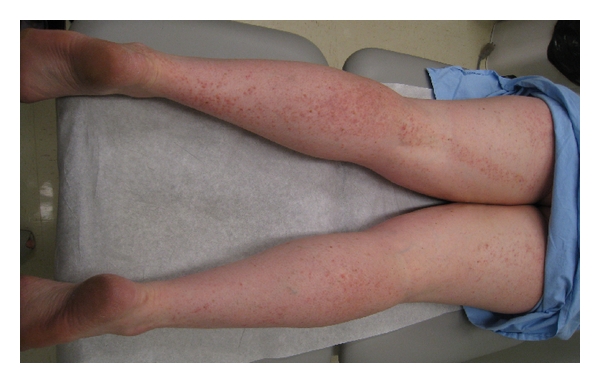
Posterior lower limbs demonstrating segmental linear plaques of warty papules on a background of less prominent hyperkeratotic papules and plaques.

**Figure 2:**
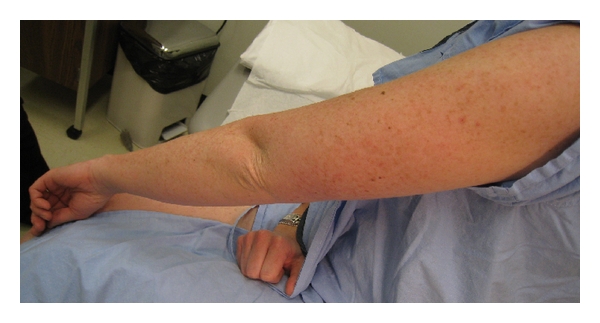
Left lateral upper limb demonstrating diffuse hyperkeratotic papules and plaques.

**Figure 3:**
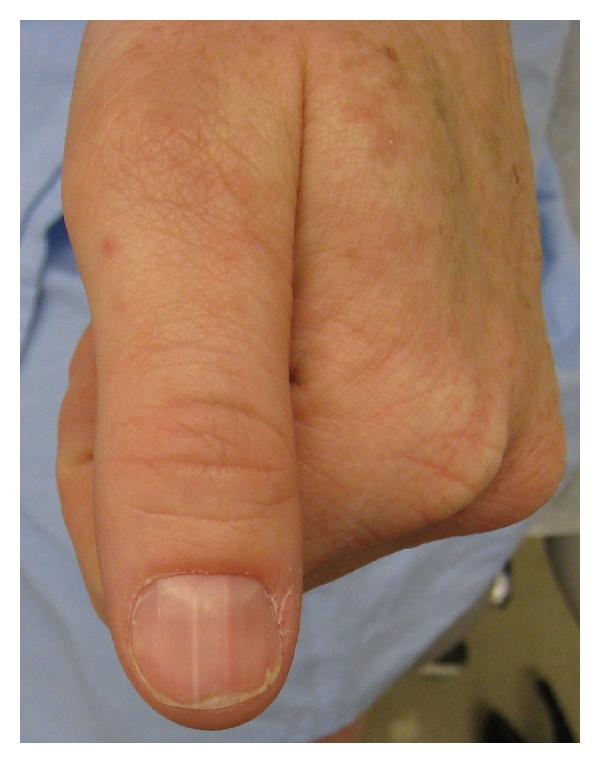
Left thumbnail demonstrating longitudinal erythronychia and distal nicking of the nail plate.

Examination revealed hundreds of erythematous to light brown scaly 2-3 mm diameter papules involving the head and neck, trunk, and extremities. These formed large linear plaques which were more prominent on the left half of the body and followed Blaschko lines (Figures 1 and 2). Similar acrokeratosis verruciformis-like warty papules as well as larger verrucous papules and plaques were noted on the dorsal hands and periungal areas. Several fingernails demonstrated longitudinal erythronychia and distal nicking of the nail plate (Figure 3). 

**Figure 4:**
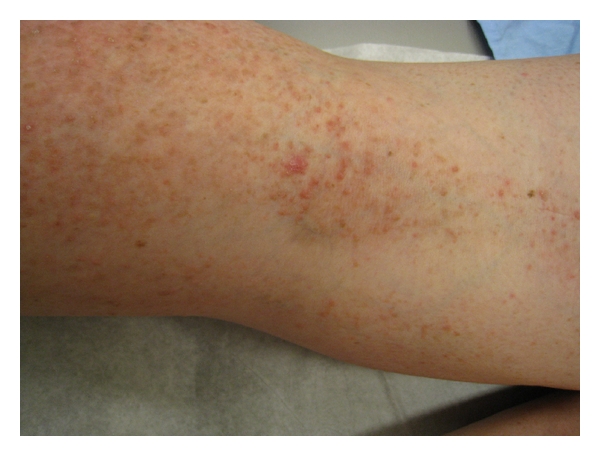
Left popliteal fossa demonstrating a shiny telangiectatic papule within a linear collection of keratotic papules in the left popliteal fossa.

**Figure 5:**
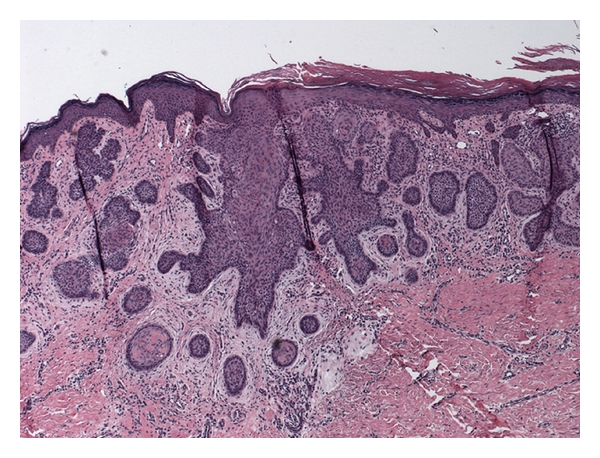
Histopathology of papule in left popliteal fossa consistent with nodular basal cell carcinoma.

**Figure 6:**
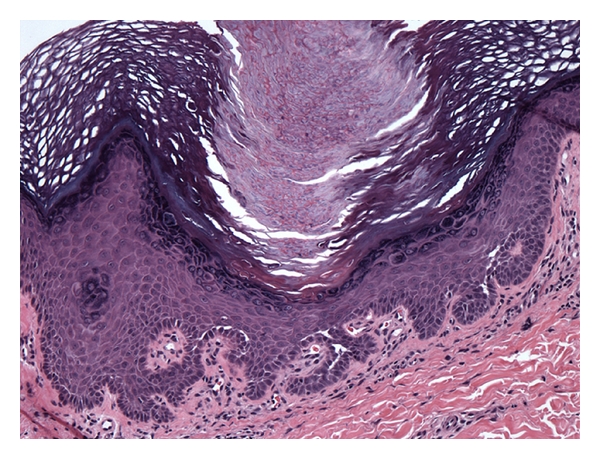
Histopathology demonstrating characteristic Darier's pathology of suprabasal acantholysis and dyskeratosis.

**Figure 7:**
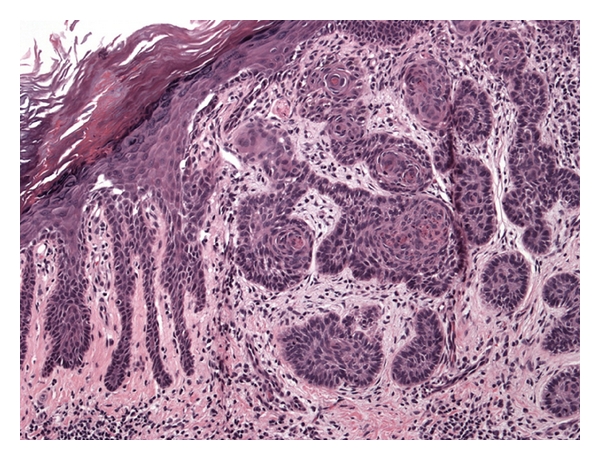
Histopathology demonstrating nodular basal cell carcinoma surrounded by characteristic suprabasal acantholysis and dyskeratosis seen in Darier's disease.

An erythematous shiny telangiectatic papule measuring 7 mm in diameter was noted within a linear collection of papules in the left popliteal fossa (Figure 4). A biopsy of the papule confirmed this to represent a nodular basal cell carcinoma (Figure 5) surrounded by characteristic suprabasal acantholysis and dyskeratosis seen in DD (Figures 6 and 7).

## 3. Discussion

We present a case of DD with well-defined segmental involvement superimposed on a milder, diffuse manifestation. The DD case is unusual due to both the distribution of the disease as well as the presence of basal cell carcinoma (BCC). 

Over 50 cases of segmental DD have been reported in the last 100 years and have been variably referred to as localized, zosteriform, linear, segmental, or unilateral. In 1997, Happle proposed a categorization of mosaic forms of autosomal dominant skin disorders [3] that includes two forms of segmental DD. Type 1 segmental manifestation presents as distinct lesions of equal severity to a nonmosaic presentation on a background of normal skin. It is believed that type 1 represents an early postzygotic mutation resulting in heterozygosity. The type 2 segmental form manifests as well-defined areas of DD occurring on the background of a less severe nonmosaic phenotype. The segmental areas of involvement can present unilaterally or as coexisting bands of either excessive or absent involvement. This latter phenotypic presentation is a result of twin spotting. Our patient's presentation is consistent with the type 2 segmental form. Genetically, type 2 segmental DD is a result of a heterozygous germline mutation compounded by a postzygotic mutation, such as mitotic recombination, nondisjunction, or deletion that leads to a homozygous or hemizygous population of cells for the underlying mutation. To date, 8 cases of type 2 segmental DD have been described in the literature [4–11] (Table 1).

Additionally, this case is unusual due to the presence of BCC in an area receiving minimal sun exposure. Given the patient's young age and the lack of significant risk factors for non-melanoma skin cancer (NMSC), the development of BCC is unusual and raises suspicion of a link between DD and cutaneous malignancy. A literature review was undertaken to identify any association between DD and BCC or any other cutaneous malignancy. The review revealed that NMSC occurring in DD has rarely been described. Since 1981, 13 cases of NMSC in patients with DD have been reported: 7 squamous cell carcinomas (SCC), 5 BCC, and 1 adenexal tumor. Of the 5 cases of BCC, 2 of the cases had previously received grenz-ray therapy and/or superficial radiotherapy [12, 13], while no risk factors were associated with the other 3 cases [13–15] (Table 2). 

While no molecular link between DD and BCC has been described, the imbalance of cellular survival and apoptosis due to the DD mutation or other genodermatosis may contribute to the presentation. Darier's disease is caused by a loss-of-function mutation in the ATP2A2 that leads to a disruption of Ca^2+^ homeostasis within the keratinocytes. A decreased SERCA activity leads to an upregulation of the transient receptor potential canonical 1 Ca channel that increases cell proliferation and resistance to apoptosis [16]. Additionally, it has been demonstrated that patients with DD have reduced expression of the antiapoptotic proteins Bcl-2 and Bcl-XL [17] which may activate apopotosis and lead to increased cell turnover. Further, alteration of ATP2A2 gene has been reported in the development of various other human carcinomas including colon and lung cancers [18]. Finally, a genetic predisposition to cancer in a related genodermatosis, Hailey-Hailey disease, has been demonstrated in murine models. Specifically, mice were bred with a mutated ATP2C1 gene leading to a similar loss in the golgi Ca^2+^ pump as in DD. The aged heterozygous mice demonstrated an increased incidence of malignancy. It was proposed by Okunade et al. that the loss of the golgi Ca^2+^ pump lead to Golgi stress and expansion which increased apoptosis and ultimately causes a genetic predisposition to cancer [19].

Although the literature is scarce and our understanding of the relationship between carcinomas and DD is developing, the case presented illustrates a possible association between DD and BCC.

## Figures and Tables

**Table 1:** Summary of the literature search of type 2 segmental cases.

	Chester and Brown [4]	Esche et al. [5]	Happle et al. [6]	Itin et al. [7]	Itin and-Happl [8]	Yusuf et al. [9]	de la Torre Fraga [10]	Rodríguez-Pazos et al. [11]	Presented
Age	25	53	45	52	17	12	24	50	34
Sex	Female	Male	Male	Female	Male	Male	Female	Male	Female
Side of involvement and areas of segmental distribution	Right side involving: Shoulder, chest, abdomen, retroauricular, and intraauricular	Face and trunk	Left side involving: retroauricular, scapular, pectoralis major, and lumbar regions	Right side involving: arm and leg	Twin spot phenomenon involving: back	N/A	N/A	Twin spot phenomenon	Left side involving: head, neck, trunk, and extremities

**Table 2:** Summary of the literature review of DD cases with BCC.

	Case 1 [12]	Case 2 [14]	Case 3 [13]	Case 4 [13]	Case 5 [15]	Case 6	Average Presentation
Age	42	38	47	48	36	34	55
Sex	Male	Male	Male	Male	Female	Female	M2.1 : F1
Tumor and Site	(a) BCC Leg (b) BCC Neck (c) BCC Arm	(a) BCC left lower eyelid (b) BCC left forehead	(a) BCC cheek (b) Multiple BCC on face chest and back (4 years post-grenz-rays)	(a) BCC ear (b) BCC scalp (c) BCC supraclavicular fossa (d) BCC sacrum	BCC cheek	BCC popliteal fossa	70% head 25% trunk 5% penis, vulva, or perianal skin
